# Adherence to dietary recommendations for preschoolers: clinical trial with teenage mothers

**DOI:** 10.1590/S1518-8787.2016050006622

**Published:** 2016-11-24

**Authors:** Betina Soldateli, Alvaro Vigo, Elsa Regina Justo Giugliani

**Affiliations:** I Departamento de Nutrição. Faculdade de Medicina. Universidade Federal do Rio Grande do Sul. Porto Alegre, RS, Brasil; II Departamento de Estatística. Instituto de Matemática e Estatística. Universidade Federal do Rio Grande do Sul. Porto Alegre, RS, Brasil; III Departamento de Pediatria. Faculdade de Medicina. Universidade Federal do Rio Grande do Sul. Porto Alegre, RS, Brasil

**Keywords:** Child, Preschool, Child Nutrition, Food Habits, Recommended Dietary Allowances, Food and Nutrition Education

## Abstract

**OBJECTIVE:**

To assess the effect of educational dietary intervention offered in the child’s first year of life, as well as teenage mothers and grandmothers in carrying out the dietary recommendations at four to seven years.

**METHODS:**

Randomized clinical trial initiated in 2006, in Porto Alegre, RS, involving 323 teenage mothers and grandmothers who cohabited. The intervention consisted of six counseling sessions on breastfeeding and healthy complementary feeding. The first session occurred in the maternity ward and the other ones in the households of mothers at seven, 15, 30, 60, and 120 days of the child’s life. The information about the child’s diet were obtained on a monthly basis in the first six months, every two months in the second half-year, and at four to seven years, using a food frequency questionnaire. To assess the adequacy of food consumption to the recommendations from the Ministry of Health, we elaborated a score system that would reflect the compliance with the Ten Steps for Healthy Toddlers from 2 to 10 Years. The average scores of intervention and control groups were compared using the t-test.

**RESULTS:**

Low adherence to recommendations on child nutrition was found in the study population, with no difference in implementation the steps between the groups. The score on the compliance with the steps was similar in both groups (9.6 [SD = 1.63] and 9.3 [SD = 1.60] in the intervention and control groups, respectively) and no influence of the cohabitation with the grandmother was found.

**CONCLUSIONS:**

Educational dietary intervention in the first four months of the child’s life for teenage mothers and grandmothers had no effect on the compliance with the recommendations at four to seven years of the child’s life.

## RESUMO

**OBJETIVO:**

Avaliar o efeito de intervenção alimentar educativa oferecida, no primeiro ano de vida da criança, a mães adolescentes e avós maternas, no cumprimento das recomendações alimentares aos quatro a sete anos.

**MÉTODOS:**

Ensaio clínico randomizado iniciado em 2006, em Porto Alegre, RS, envolvendo 323 mães adolescentes e avós maternas, quando em coabitação. A intervenção consistiu em seis sessões de aconselhamento sobre aleitamento materno e alimentação complementar saudável. A primeira sessão ocorreu na maternidade e as demais, nos domicílios das mães aos sete, 15, 30, 60 e 120 dias de vida da criança. As informações sobre alimentação da criança foram obtidas mensalmente nos primeiros seis meses de vida, a cada dois meses no segundo semestre, e aos quatro a sete anos, por meio de questionário de frequência alimentar. Para avaliar a adequação do consumo alimentar às recomendações do Ministério da Saúde, elaborou-se um sistema de escore que refletisse o cumprimento dos Dez Passos Para Uma Alimentação Saudável Para Crianças de 2 a 10 Anos. As médias dos escores dos grupos intervenção e controle foram comparadas por meio do teste t.

**RESULTADOS:**

Houve baixa adesão às recomendações sobre alimentação infantil na população estudada, sem diferença no cumprimento dos passos entre os grupos. O escore relativo ao cumprimento dos passos foi semelhante nos dois grupos (9,6 [DP = 1,63] e 9,3 [DP = 1,60] nos grupos intervenção e controle, respectivamente) e não houve influência da coabitação com a avó materna.

**CONCLUSÕES:**

Intervenção alimentar educativa nos primeiros quatro meses de vida da criança para mães adolescentes e avós maternas não teve efeito no cumprimento das recomendações alimentares aos quatro a sete anos de vida.

## INTRODUCTION

The education of an individual’s eating habits occurs under the influence of a complex network of genetic and environmental factors, starting in intrauterine life^2^. The first years of life are very important for the establishment of these habits, with repercussions on the health of the individual in future stages^16^ ^,^ ^20^. Despite this, the feeding of young children in general is inadequate, as shown by studies in several countries, including Brazil^5^ ^,^ ^8^. Nationwide survey found that Brazilian children between six and 59 months present low consumption of vegetables and meats and high intake of sodas, fried foods, snacks, and candies^5^.

This scenario indicates the urgent need for interventions aiming to improve the eating habits of children in the first years of life, namely, promoting exclusive breastfeeding (EBF) in the first six months and, thereafter, to introduce healthy complementary foods, maintaining breastfeeding for two years or more^a^. As a contribution to this demand, we conducted a randomized clinical trial involving teenage mothers and grandmothers, when they cohabited. The choice of this population considered that being a teenage mother and cohabiting with the grandmother may be risk factors for shorter duration of breastfeeding (BF)^9^. The intervention, which occurred in the first four months of the children’s lives, was successful in increasing the duration of the EBF and the prevalence of BF at the end of the first year of life, as well as delaying the introduction of complementary foods^4^ ^,^ ^13^ ^,^ ^14^.

Bearing these results in mind, we decided to research the eating habits of the children involved in the clinical trial, in preschool stage, since studies show association between increased duration of BF and later introduction of complementary feeding, as well as more duration of BF and EBF and better eating habits in preschool^6^ ^,^ ^10^ ^,^ ^12^. This article, therefore, aimed to assess the effect of an intervention for BF and healthy complementary feeding offered, during the first year of the child’s life, to teenage mothers and grandmothers who cohabited, on the eating habits of children at the age of four to seven years.

## METHODS

This study is a continuation of a randomized clinical trial conducted between 1 May 2006 and 30 January 2008, involving 323 teenage mothers and their mothers (grandmothers of the children), when they cohabited. The sample calculation is described in other publications^13^ ^,^ ^14^. The teenagers were recruited in the maternity ward of the Hospital de Clínicas of Porto Alegre (HCPA). It is a public general hospital, certified in the Baby-friendly Hospital Initiative, which makes around 3,000 deliveries per year.

Daily, mothers who filled the following inclusion criteria were identified: age less than 20 years; living in the municipality of Porto Alegre; with healthy babies whose birth weight was equal to or higher than 2,500 g; and that had begun breastfeeding. We did not include in this study mothers of twins and those that, due to problems of their own or of their babies, could not stay in the accommodation, as well as the teens who lived with the mothers-in-law, to avoid their possible influence. Once identified, the teenagers were randomly allocated to the intervention or to the control group, in two blocks. To verify the influence of the grandmother on the intervention, we stipulated that teenagers who lived with their mothers would form half of the sample.

The intervention consisted of six counseling sessions in breastfeeding and healthy complementary feeding: the first one in the maternity ward, in the second or third day after delivery, and the other ones in the households of mothers at seven, 15, 30, 60, and 120 days of the child’s life. When the mother and grandmother did not cohabit, only the teenager received the intervention. The sessions were always conducted by the same professional from a staff with two nurses, one nutritionist, and one pediatrician, of which three were consultants in milk by the International Board of Lactation Consultant Examiners. The principles of breastfeeding advice recommended by the World Health Organization (WHO) were followed, i.e., dialogue among mothers, grandmothers, and professionals addressing various aspects of BF and food supplement, considering the particularities of each mother or grandmother^b^. Support materials were used, such as information booklets produced especially for the research. All mothers, regardless of the group to which they were allocated, received standard care from the maternity.

In the maternity ward, the intervention was made separately for mothers and grandmothers who cohabited; in households, they were given joint sessions of counseling. In these sessions, the advices passed on in the maternity were reinforced and the difficulties faced by the mothers regarding the feeding of the child were discussed. At four months, the intervention emphasized the introduction of healthy complementary feeding starting from six months, according to the guidelines of the Food Guide for Children under Two years old^c^. On this occasion, booklets with guidelines on healthy complementary feeding and timely manner were distributed.

Data were collected in March 2015. At the maternity ward, the mothers and the grandmothers who cohabited, after agreeing to participate in the research, signed an informed consent form, were interviewed separately to obtain data and socioeconomic aspects related to prenatal, childbirth, and prior experience with breastfeeding. Information about the child’s diet in the first year of life were obtained on a monthly basis in the first six months and every two months in the second half-year, by telephone interview with the mother, or home visits in case telephone contact was impossible. The following assessment occurred when the children were between four and seven years of age, at the Clinical Research Center of the HCPA or in the households, in case the mother and the child could not go to the center. On that occasion, the mothers were interviewed to obtain information on the current socioeconomic characteristics and about the diet of the children, using a food frequency questionnaire. Data collection was performed by researchers who did not know to which group (experimental or control) the mothers belonged.

Collected data were stored on computer by double typing, using the Excel^®^ program, with later validation. For statistical analysis, we used the Statistical Analysis System program (SAS 9.4). The statistical analyses were based on the “intention-to-treat” concept. To compare the proportions, we applied the Pearson Chi-square or Fisher exact tests.

To assess the adequacy of food consumption to the recommendations from the Ministry of Health (MH), i.e., the compliance with “Ten Steps for Healthy Toddlers from 2 to 10 Years^d^”, we elaborated a system of scores that scored the steps as they were fulfilled: two points for the step completed, one point to the step partially completed, and zero points for the step not completed (Box 1). Step 10 was not included in the analysis as it refers to physical activity. Thus, the score of each child could range from zero to 18 points. The average scores of intervention and control groups were compared using the Student’s t-test. The significance level adopted was 5% (p ≤ 0.05).

**Box 1:** The “Ten Steps for Healthy Toddlers from 2 to 10 Years” and criteria used for the score of each step.

Steps	Score
1. To offer a variety of foods, distributing them in at least three meals and two snacks a day. It is important for the child to eat slowly.	2 = *Variety*: consumes ≥ 4 food groups, 5 x/week; *Frequency*: breakfast/lunch/dinner, and ≥ 2 snacks; *Eats slowly*: yes 1 = any situation other than the equivalent to the score 2 and 0 0 = *Variety*: consumes ≥ 2 food groups, 5 x/week; *Frequency*: do not have breakfast or lunch or dinner; *Eats slowly*: no
2. To include daily foods such as cereals (rice, corn), tubers (potatoes), roots (manioc, cassava, yuca), breads and pastas, distributing these foods in children’s meals and snacks throughout the day.	2 = consumes cereals or tubers/roots > 5 x/week ≥ 1 x/day 1 = any situation other than the equivalent to the score 2 and 0 0 = consumes cereals or tubers/roots > 2 x/week ≥ 1 x/day
3. To offer vegetables in the two main meals of the day; also provide, daily, two fruits as desserts and snacks.	2 = consumes vegetables and fruits > 5 x/week, 2 x/day 1 = any situation other than the equivalent to the score 2 and 0 0 = consumes vegetables and fruits > 5 x/week, 2 x/day
4. To offer rice and beans every day or at least five times a week. Shortly after the meal, offer half a glass of fruit juice or natural fruit that is a source of vitamin C.	2 = consumes beans and rice ≥ 5 x/week; natural juice after the meal 1 = any situation other than the equivalent to the score 2 and 0 0 = consumes beans and rice < 3x/week; do not receive juice
5. To offer milk or dairy products (yogurt and cheese) three times a day. If the child is still being breastfed, there is no need to offer other milk. Meat, poultry, fish, or eggs should be part of the child’s main meal.	2 = consumes milk/dairy products > 5 x/week, 3 x/day; meat and/or eggs > 5 x/week ≥ 1 x/day 1 = any situation other than the equivalent to the score 2 and 0 0 = consumes milk/dairy products ≤ 5 x/week, ≤ 1 x/day; meat and/or eggs up to 3 x/week
6. To avoid fatty and fried foods; prefer baked, grilled, and stewed foods. To remove the visible fat from meats and poultry skin before preparation.	2 = consumes fried foods ≤ 1 x/week; most frequent preparation: baked, grilled, stewed; remove fat from the meat before cooking 1 = any situation other than the equivalent to the score 2 and 0 0 = consumes fried foods > 3 x/week; most frequent preparation: fried food; do not remove fat from meat
7. To avoid soft drinks and industrialized juices or foods with too much sugar (candy, chocolates, cookies), snacks and other goodies on a daily basis.	2 = consumes soft drinks/juices, candies, and snacks ≤ 1 x/week 1 = consumes soft drinks/juices, candies, and snacks 2 - 3 x/week 0 = consumes soft drinks/juices, candies, and snacks > 3 x/week
8. To decrease the amount of salt in the food. To avoid ready-made seasoning, canned foods, salted meats, and sausages.	2 = does not consume ready-made seasoning; consumes canned foods and sausages ≤ 1 x/week; food prepared with less salt for the child. 1 = any situation other than the equivalent to the score 2 and 0 0 = always consumes ready-made seasonings; canned food and sausages ≥ 3 x/week
9. To encourage the child to drink at least four glasses of water during the day, preferably in between meals.	2 = drinks at least 4 cups (250 ml) of water per day (= 1,000 ml) 1 = drinks from 2 to 3 cups (250 ml) of water per day (500-750 ml) 0 = drinks ≤ 1 cup (250 ml) of water per day
10. In addition to the diet, regular physical activity is important to maintain the weight and a healthy life.	* Not assessed

This study was conducted in accordance with the Guidelines for Health Research (Ordinance 01/88 National Health Congress, supplemented by Resolution 466/2012). The research was approved by the Scientific Committee and Research Ethics Committee in Health of the HCPA and by *Plataforma Brasil* (120249). The clinical trial was registered in ClinicalTrials.gov, under number NCT00910377.

## RESULTS

The Figure shows the flowchart of participation of individuals involved in the clinical trial since the recruitment until the last assessment, when the child was between four and seven years. Of the 323 mothers who initiated the study, 207 (64.1%) were located and attended the last assessment. The losses of the study occurred for no location of mothers/families (n = 91), refusal to remain in the study (n = 23), separation between mother and baby (n = 1), and death of the mother (n = 1).

**Figure:**
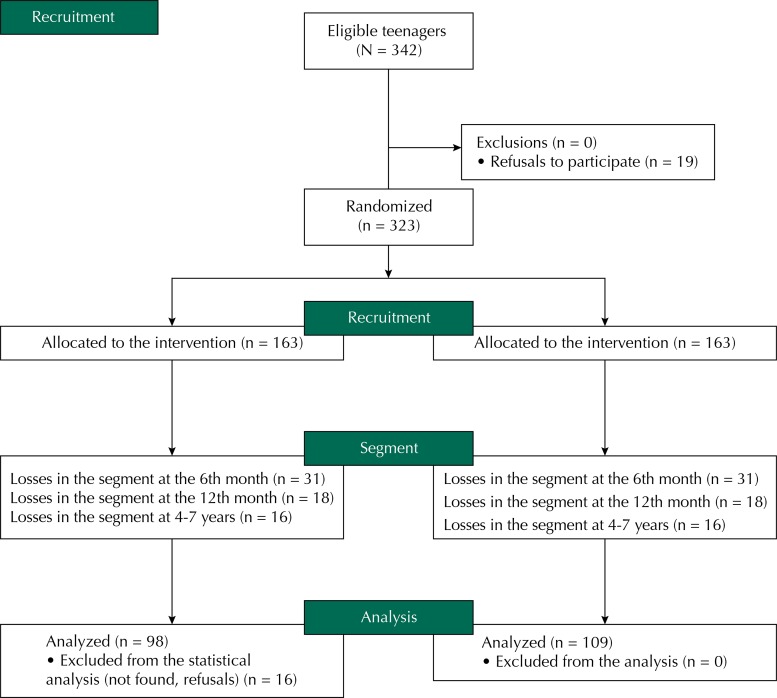
Flowchart of the participants in the clinical trial since the recruitment until the last assessment of the child, at 4-7 years.

The comparison of the characteristics of individuals who participated in the last assessment showed an imbalance between intervention and control groups: the intervention group had higher proportion of cohabitation with the grandmother at the time of the intervention (65.3% *versus* 48.6% in the control group; p = 0.023) and of mothers with ≥ 8 years of schooling in the last assessment (80.9% *versus* 67.3% in the control group; p = 0.044) and lower average age of the children (5.8 years *versus* 6.3 years; p = 0.044). In relation to age group, 58 children (59.2%) were between four and five years in the intervention group and 17 (15.6%) in the control group; in between six and seven years, 40 (40.8%) and 92 (84.4%) belonged to the intervention and control groups, respectively.

Generally, we observed low adherence to recommendations on child nutrition in the study population, with no difference between the groups regarding the compliance with the steps. The score concerning the compliance with the steps ranged from 5.2 to 13.8 and their averages were similar in the intervention and control groups. No significant difference was found in the scores between the two groups after the sample stratification according to the cohabitation with the grandmother (Table 1). Table 2 shows the percentage of compliance with the “Ten Steps for Healthy Toddlers from 2 to 10 years.” With the exception of step 9, concerning the intake of water, which was not fulfilled by any child, most children fulfilled partially. Less than 1.0% of children fulfilled the steps 5 (consumption of milk and dairy products, meat, and eggs), 7 (low consumption of unhealthy foods), and 8 (amount of salt in food preparation). Step 10 was not included in the analysis as it refers to physical activity.

**Table 1:** Scores of compliance with “Ten Steps for Healthy Toddlers from 2 to 10 Years,” by group and stratification by cohabitation with the grandmother.

Group	Intervention		Control		p
	
	Score (average)	SD	Score (average)	SD	
Total sample (n = 207)	9.6	1.63	9.3	1.6	0.223
Mothers and grandmothers in cohabitation (n = 113)	9.4	1.67	9.2	1.42	0.483
Mothers and grandmothers without cohabitation (n = 94)	9.8	1.55	9.3	1.76	0.221

**Table 2:** The percentage of compliance with the “Ten Steps for Healthy Toddlers from 2 to 10 years” in intervention and control groups.

	Group	Compliance with the steps			Total	p

		Total	Partial	No		
		
		n (%)	n (%)	n (%)		
Step 1	Intervention	10 (10.2)	87 (88.8)	1 (1.0)	98	
	Control	10 (9.3)	90.7	0 (0.0)	108	0.724
Step 2	Intervention	37 (37.8)	60 (61.2)	1 (1.0)	98	
	Control	39 (35.8)	70 (64.2)	0 (0.0)	109	0.663
Step 3	Intervention	10 (10.2)	80 (81.6)	8 (8.2)	98	
	Control	16 (14.7)	86 (78.9)	7 (6.4)	109	0.580
Step 4	Intervention	21 (21.4)	71 (72.4)	6 (6.1)	98	
	Control	22 (20.2)	76 (69.7)	11 (10.0)	109	0.582
Step 5	Intervention	1 (1.0)	94 (95.9)	3 (3.0)	98	
	Control	0 (0.0)	102 (93.6)	7 (6.4)	109	0.309
Step 6	Intervention	24 (24.5)	71 (72.4)	3 (3.0)	98	
	Control	25 (22.9)	79 (72.5)	5 (4.6)	109	0.864
Step 7	Intervention	2 (2.0)	80 (81.6)	16 (16.3)	98	
	Control	0 (0)	87 (79.8)	22 (20.2)	109	0.311
Step 8	Intervention	1 (1.0)	96 (98.0)	1 (1.0)	98	
	Control	0 (0)	105 (96.3)	4 (3.7)	109	0.278
Step 9	Intervention	-	18 (18.6)	79 (81.4)	97	
	Control	-	22 (20.4)	86 (79.6)	108	0.743

Linear regression analysis was performed to assess the difference among the averages of the overall score of intervention and control groups, in model adjusted by maternal education variables, cohabitation with the grandmother, age of the child, and breastfeeding time. The difference between the averages was 0.21 (95%CI -0.30–0.74), with no statistical significance.

## DISCUSSION

This study is unprecedented; it is the first clinical trial that tested nutritional interventions focusing exclusively on teenage mothers. Due to emotional immaturity and intense process of physical, psychological, and social changes, motherhood in adolescence can compromise the care of the baby, including their food and, consequently, their nutritional status. Therefore, it is essential to offer adequate support in the area of maternal and child health to these mothers, considering the peculiarities related to adolescence^e^. This study idealized the intervention after conducting focus group with teenage mothers, developing educational materials with attractive engravings and vocabulary for this audience. In addition, as teenage mothers commonly live with their own mothers, this aspect has also been included in the intervention, manner unprecedentedly, involving the grandmothers.

Our initial hypothesis was that the intervention could have a positive effect on the eating habits of children at the age of four to seven years, improving the quality of their diet, despite the intervention being conducted in the first year of life. This hypothesis was ventilated bearing in mind the positive impact of the intervention on the duration of the BF and EBF and at the time of the introduction of complementary feeding. Studies show positive effects of longer duration of BF and EBF on eating habits in preschoolers^6^ ^,^ ^10^ ^,^ ^12^. However, the results of this study do not confirm our hypothesis, as no differences were found in the diet quality of children aged four to seven years, assessed by the compliance with the Ten Steps for Healthy Toddlers from 2 to 10 Years, recommended by the MH. This result is contrary to the findings of a study conducted with similar Brazilian population, whose intervention, also held in the first year of life, increased the consumption of vegetables and fruits and the diversity of the diet in children aged three to four years^20^. However, the study differs from ours for having held a longer-lasting intervention (until the end of the first year of life), by the fact that the population is composed mostly by adult women and do not involve the grandmothers. However, curiously, that same intervention lost the effect when the children’s diet was reassessed at seven to eight years of age^15^. A similar result occurred in the study by Scheiwe et al.^17^, in London. The intervention, which consisted of monthly meetings from three to 12 months after the delivery, had positive effect on the consumption of fruits and vegetables at 18 months, but little effect on the diet quality at the age of four. These findings reinforce the argument that long periods between the intervention and the outcome are unfavorable, as occurred in our study, particularly when the outcome (eating habits) suffers the influence of numerous factors.

Another factor that may have contributed to the lack of impact of the intervention on the quality of the children’s diet was the fact that the supplementary feeding was addressed in a single moment, at four months of the child’s life, without later reinforcement sessions, although a booklet with the proposed recommendations for a healthy diet was distributed. Recent review of the literature on the strategies to promote healthy and successful complementary feeding pointed out that the messages with greater nutritional impact are concise, easy-to-remember, relevant, few in number, and intended for all caregivers of babies and young children. In addition, the key messages are passed on in several occasions, by various channels of intervention, by multiple health professionals, in health services and within the community^11^. According to the WHO, interventions need to be sustained over time, integrated, as well as directed to multiple risk factors^f^.

It is important to document that, regardless of the group, low adherence to national recommendations for a healthy diet was found. Not even half the children completed the step 2, concerning the daily consumption of cereals, which was the step with better compliance index. Some steps were completed by 2.0% of the children or less (steps 5, 7, and 8). These steps are very important because they concern the consumption of proteins of high biological value (step 5) and consumption of unhealthy and high-sodium foods (steps 7 and 8). It should be noted that about 20% of the children consumed soft drinks, candies, and snacks (step 7) three times or more per week.

In addition, no child completed the step 9, concerning the intake of at least one liter of water per day. According to the Institute of Medicine, the adequate water intake for children from four to eight years is 1.7 liter. This value refers to the total fluid intake, considering the water contained in foods, liquids, and pure water^19^. Probably, the children are getting the recommended amount of water in the form of soft drinks and juices, as shown in another publication with the same sample^18^. The daily consumption of artificial juice by the population studied, in both groups, was very high (control: 85.3%; intervention: 73.5%). This finding is worrying, considering that the regular intake of sugary drinks increases the risk of overweight in childhood^7^.

Some steps are indicators of an unhealthy diet (steps 6, 7, and 8), and concern the consumption of foods with a high amount of fat, sugar, and salt, which had as definition of ideal consumption the maximum frequency of once a week. However, most children consumed such foods from two to three times a week. These foods have a high energy density and are poor in nutrients; the consumption beyond the recommended indicates that the children, in addition to increasing the consumption of healthy foods, need to decrease the consumption of unhealthy foods.

The low quality of the diet found in this study is in accordance with representative data on the population of Brazilian children from two to five years, which have low consumption of vegetables and meats, and high intake of sodas, fried food, snacks, and candies, as well as with other studies on preschoolers^5^ ^,^ ^10^ ^,^ ^20^.

Surveys conducted in several cultural contexts indicate that grandmothers have influence and involvement on the child’s diet^1^. A qualitative study with teenage mothers and grandmothers held in Baltimore, USA, showed that most grandmothers studied played dominant role in deciding what the child should eat as well as in the moment of the introduction of solid food^3^. Thus, it was expected that the inclusion of grandmothers who cohabited with their daughters could have some influence on the results. That did not happen because the intervention did not significantly modify the compliance with the ten steps, regardless of the participation of the grandmother in the intervention. However, it is important to note that, although 56.0% of the mothers/children assessed cohabit with their grandmother at the time of the intervention, only 25% remained living with their grandmothers in the last evaluation. This fact may have influenced the result. However, we did not find, in the literature review, intervention studies addressing the influence of grandmothers on the eating habits of their grandchildren.

Some limitations of the study must be considered. The segment had a large number of losses (35.9%). Such losses are common in population-based studies involving young people living in peripheral areas of developing countries. We believe that the losses did not influence significantly the results, since it was kept the proportion of subjects in the intervention and control groups (47.3% and 52.7%, respectively). The losses during the 12 months of the child’s life and the last evaluation (four to seven years), despite the long break, were relatively lower than the losses during the first year, in which the contacts were more frequent. In fact, the number of children in the control group at four to seven years was higher than at 12 months. This probably was due to the inclusion of social networks as one strategy to search the families for the last assessment, which allowed the rescue of some families that were lost in the first 12 months.

Another possible limitation is the wide age range (four to seven years) in the assessment segment, result of the combination of the long period of recruitment of the sample (approximately two years) and search for the last assessment (about 10 months), and the lack of definition of a particular age in the outcome. The study design (randomized clinical trial) certainly contributed to minimizing this limitation.

In conclusion, this study showed that an educational dietary intervention, offered to teenage mothers and grandmothers in the first four months of the child’s life, had no effect on the compliance with the recommendations at four to seven years of life. In addition, it confirmed the poor quality of preschoolers’ diet and the need for effective interventions. It also reinforced the WHO’s recommendation that educational interventions on child nutrition must be sustained, due to the dynamic nature of eating habits. Among the numerous factors, adolescent motherhood should certainly be considered, due to its peculiarities and the social context of this condition. In addition, the study may provide elements for the revision of the Ten Steps by MH, perfecting the recommendations for children older than two years.

## Footnotes

a World Health Organization. The optimal duration of exclusive breastfeeding: report of an expert consultation. Geneva; 2001 [cited 2015 Oct 30]. Available from: http://www.who.int/nutrition/publications/optimal_duration_of_exc_bfeeding_report_eng.pdf

b World Health Organization; UNICEF. Breastfeeding counselling: a training course. Geneva; 2006 [cited 2015 Oct 30]. Available from: http://www.who.int/maternal_child_adolescent/documents/pdfs/bc_participants_manual.pdf

c Ministério da Saúde (BR); Organização Mundial da Saúde. Guia alimentar para crianças menores de 2 anos. Brasília (DF); 2005. (Série A. Normas e Manuais Técnicos). [cited 2015 Oct 30]. Available from: http://bvsms.saude.gov.br/bvs/publicacoes/guia_alimentar_criancas_menores_2anos.pdf

d Ministério da Saúde (BR). Caderneta de saúde da criança: passaporte da cidadania. 5.ed. Brasília (DF); s.d. [cited 2016 Oct 31]. Available from: http://agendaprimeirainfancia.org.br/arquivos/caderneta_saude_crianca_5ed.pdf

e World Health Organization, The Partnership for Maternal, Newborn and Child Health. Reaching child brides. Geneva; 2012. (Knowledge summary, 22). [cited 2015 Oct 30]. Available from: http://www.who.int/pmnch/knowledge/publications/summaries/knowledge_summaries_22_reaching_child_brides/en/

f World Health Organization. Report of the first meeting of the ad hoc working group on science and evidence for ending childhood obesity: 18-20 June 2014, Geneva [cited 2015 Oct 30]. Available from: http://apps.who.int/iris/bitstream/10665/ 133545/1/9789241507653 _eng.pdf?ua=1&ua=1
